# Bone healing at non-submerged implants installed with different insertion torques: a split-mouth histomorphometric randomized controlled trial

**DOI:** 10.1186/s40729-019-0194-2

**Published:** 2019-12-05

**Authors:** Yoshiyuki Amari, Adriano Piattelli, Karol Alí Apaza Alccayhuaman, Natalia Fortich Mesa, Mauro Ferri, Giovanna Iezzi, Daniele Botticelli

**Affiliations:** 1 ARDEC Academy, Ariminum Odontologica, Viale Giovanni Pascoli 67, 47923 Rimini, Italy; 2 Department of Medical, Oral and Biotechnological Sciences, University of Chieti-Pescara, Chieti, Italy; 3 Biomaterials Engineering, Catholic University of San Antonio of Murcia (UCAM), Murcia, Spain; 4 Program of Odontology, University Corporation Rafael Núñez, Cartagena de Indias, Colombia; 5 Department of Medical, Oral and Biotechnological Sciences, University of Chieti-Pescara, Chieti, Italy

**Keywords:** Bone-to-implant contact, Bone density, Dental implants, Histology, Histomorphometry

## Abstract

**Objectives:**

To evaluate histomorphometrically the healing at implants installed with standard or very low insertion torque values

**Material and methods:**

Twelve volunteer patients were recruited, and two screw-shaped titanium devices were installed in the distal segments of the mandible using insertion torque values of either < 10 Ncm or ~ 30 Ncm. The implants were left to heal in a non-submerged fashion. After 8 weeks, biopsies were retrieved, and ground sections were prepared for histological evaluation.

**Results:**

Histological slides from 11 patients were available for (*n* = 11). The new bone in contact with the implant surface was 39.3 ± 18.5% and 49.4 ± 9.4% at the < 10 and ~ 30 Ncm sites, respectively. Considering the pre-existing old bone, the total mineralized bone was 46.8 ± 22.1% at the < 10 Ncm sites and 57.0 ± 14.1% at the ~ 30 Ncm. No statistically significant differences were found.

New bone density and total mineralized bone density were 36.6 ± 8.1% and 53.0 ± 13.5% at the < 10 Ncm sites and 35.9 ± 10.0% and 52.2 ± 16.0% at the ~ 30 Ncm sites, respectively. No statistically significant differences were disclosed.

**Conclusion:**

From the data of the present study, it can be concluded that a trend of higher osseointegration was observed at the ~ 30 Ncm compared to the < 10 Ncm torque group. Nevertheless, it can be concluded that an implant installed with a very low torque may achieve a good integration.

**Trial registration:**

ClinicalTrials.gov NCT04017156; trial retrospectively registered on 12 July 2019.

## Introduction

Very high insertion torque values have been recommended when immediate loading is applied to implants [1]. However, it has been shown that similar clinical outcomes may be achieved even with insertion torque values ≤ 15 Ncm when implants are splinted together [2]. Nevertheless, in a consensus conference, insertion torque values comprised between 20 and 45 Ncm were recommended for implants immediately loaded with single crowns [3]. Moreover, in animal experiments, the highest rate of osseointegration was observed when insertion torque of 30–35 Ncm was applied [4–6].

Different insertion torque values have been tested both in human [7–9] and in animals studies [1, 4–6, 10–12]. A systematic review with meta-analyses summarized data from both animal and human studies and two groups were identified based on the insertion toque values: > 25 Ncm or < 30 Ncm [13]. No differences were found between the two groups in terms of implant survival rate or marginal bone loss. Nevertheless, in a randomized clinical study [7], after 12 months of healing, implants installed with torque ≥ 50 Ncm presented higher marginal bone loss compared to implants with torque included between 20 and 50 Ncm. Very low insertion torques have been also tested both in animals and humans. After 4 months of healing, in an experimental study in dogs [5], an osseointegration > 55% was obtained at implant installed with torque close to 0 Ncm. In a clinical study [8], 11 implants were installed with a torque < 10 Ncm. After 4 to 6 months, a reversal torque of 35 Ncm was applied and all implants showed a good stability.

However, it has to be considered that implants presenting a rotational instability during implant installation resulted in a lower survival rate compared to implants installed with higher insertion torque [14, 15].

Due to the contradictory outcomes on the influence of the torque on osseointegration and a lack of histological data in humans, there is a need of more evidences that may support the clinicians in the decision making when an unintentional low insertion torque occur at implants during the daily practice.

Hence, the aim of the present study was to evaluate histomorphometrically the healing at implants installed with standard or very low insertion torque values.

The hypothesis was that a higher bone-to-implant contact of the microimplant would be observed in the group with higher torque compared to the group with a lower torque.

## Materials and methods

### Patient selection

The Declaration of Helsinki on medical protocols and ethics was followed. The protocol was approved by the Ethical Committee of the Corporación Universitaria Rafael Núñez, Cartagena de Indias, Colombia, with protocol #04-2014 on October 8, 2014. All treatments were performed in that institution from November 2016 to October 2017. All surgical procedures and possible complications were clearly explained to each participant and a written informed consent was signed by all patients. The study was reported according to the CONSORT guidelines and registered at the ClinicalTrials.gov with the code NCT04017156.

For sample calculation, data from a dog experiment were used [5] and a sample of 12 subjects was calculated for matched pairs considering a difference in bone-to-implant contact of 10% being clinically relevant. A power of 0.8 and *α* = 0.05 were used.

The following inclusion criteria were required: (i) presence of at least two edentulous zone in the posterior segment of the mandible; (ii) ≥ 25 years of age; (iii) smoking ≤ 10 cigarettes per day; (iv) good general health; (v) no contraindication for oral surgical procedures; and (vi) not being pregnant. The following exclusion criteria were adopted: (i) presence of systemic disorders; (ii) chemotherapy or radiotherapy; (iii) smokers > 10 cigarettes per day; and(iv) previous bone augmentation procedures in the region.

### Device

**Fig. 1:**
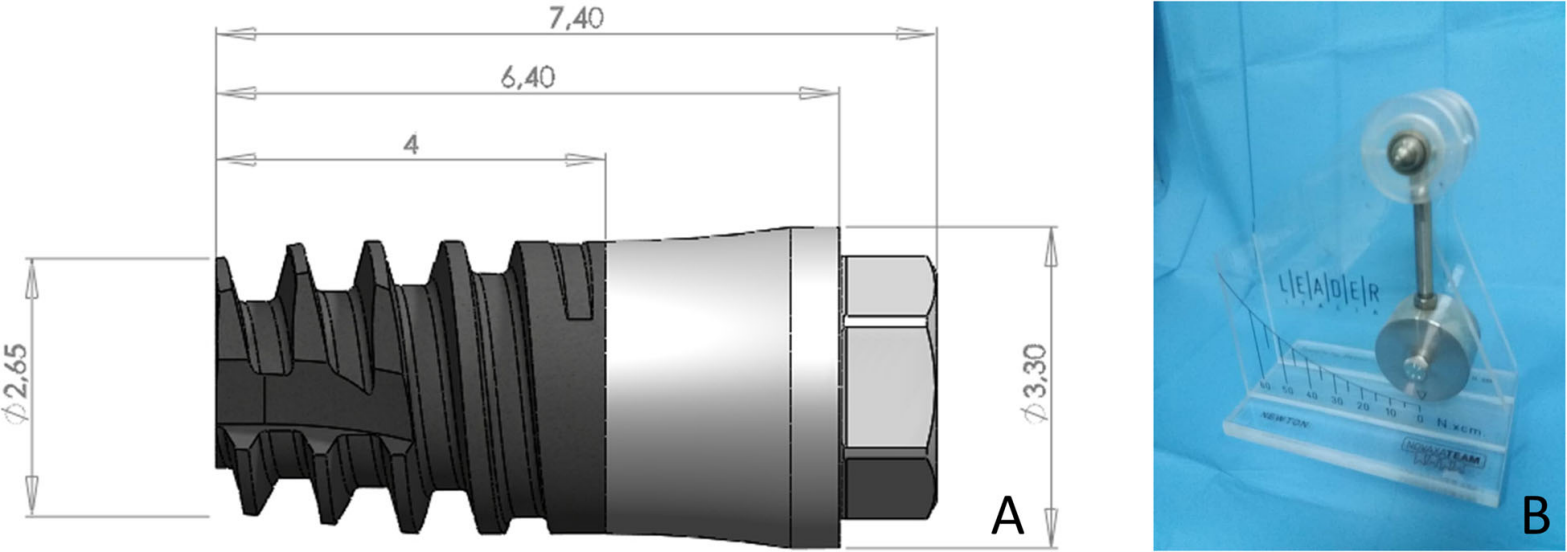
**a** Customized solid titanium screw-shaped devices used. **b** Newton-meter to measure the torque

Customized solid titanium screw-shaped devices were used (Sweden & Martina, Due Carrare, Padua, Italy). The devices had an intraosseous portion with a moderately rough surface [16] (ZirTi® surface, Sweden & Martina, Due Carrare, Padua, Italy). The intraosseous portion was 4 mm long, with a diameter of 2.65 mm at the apical aspect and 3 mm at the coronal margin. A polished neck 2.4 mm long junction represented the transmucosal portion of the implant (Fig. 1a).

### Randomization

Each patient received two mini-implants, installed in recipient sites prepared either to reach torque of < 10 Ncm or ~ 30 Ncm. The two recipient sites were selected prior the surgery, while the type of site preparation was randomly decided. The randomization was performed electronically (randomization.com) by a researcher neither involved in the selection of the patients nor in the installation of the devices (DB). Sealed opaque envelopes were prepared and opened at the time of surgery.

### Clinical procedures

**Fig. 2:**
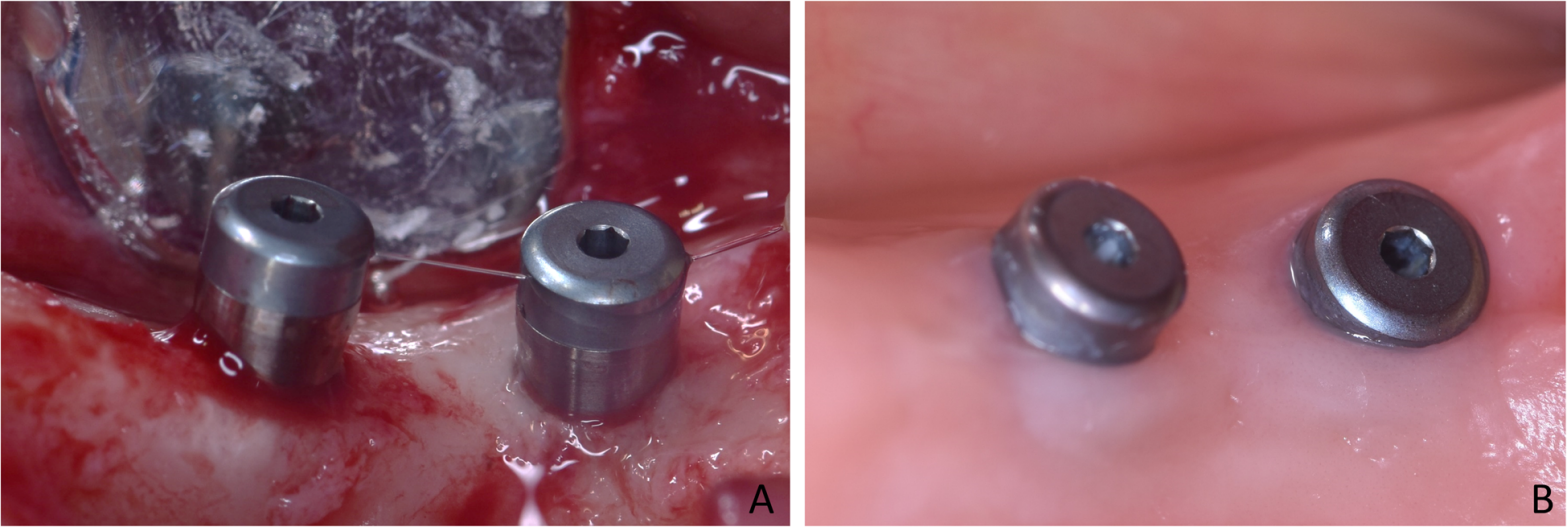
Two devices installed, one using insertion torque of < 10 Ncm (**a**) and one of ~ 30 Ncm (**b**)

The surgical procedures were performed by an expert surgeon (MF). After the injection of local anesthesia, small crestal and releasing incisions were performed in the distal segments of the mandible, and small full-thickness muco-periosteal flaps were elevated. The test sites (< 10 Ncm) were over-prepared with drills of larger diameter compared to those used at the standard sites (~ 30 Ncm). The recipient sites at the ~ 30 Ncm were prepared up to a diameter of 1.8 mm to a depth of 6 mm and the coronal 2 mm were enlarge to 2.4 mm. The < 10 Ncm sites were prepared to a depth of 6 mm with a lanceolate drill to with a maximum diameter of 2.4 mm. The coronal region was then enlarged to 2.8 mm. All site preparations were performed deeper compared to the length of the implant so that the implant apex could not reach the bottom of the osteotomy. This, in turn, means that the final torque was produced by the lateral pressure against the bone walls, as described in a previous animal experiment [4]. The mini-implants were subsequently installed (Fig. 2a), and the final insertion torque was measured with wrench calibrated on a newton-meter (Newton; Leader Italia, Cinisello Balsamo, MI, Italy) (Fig. 1b). A cover screw was placed on the top of the mini-implants, and the flaps were sutured allowing a non-submerged healing.

### Maintenance

Antibiotics (amoxicillin 875 mg/clavulanic acid 125 mg twice a day for 6 days) and non-steroidal anti-inflammatory drugs as needed (ibuprofen 400 mg) were prescribed. Mouth rinses with 0.12% chlorhexidine three times a day for 10 days were also recommended.

After 7 days, the sutures were removed, and the patients were recalled every 2 weeks after surgery.

### Biopsies

**Fig. 3:**
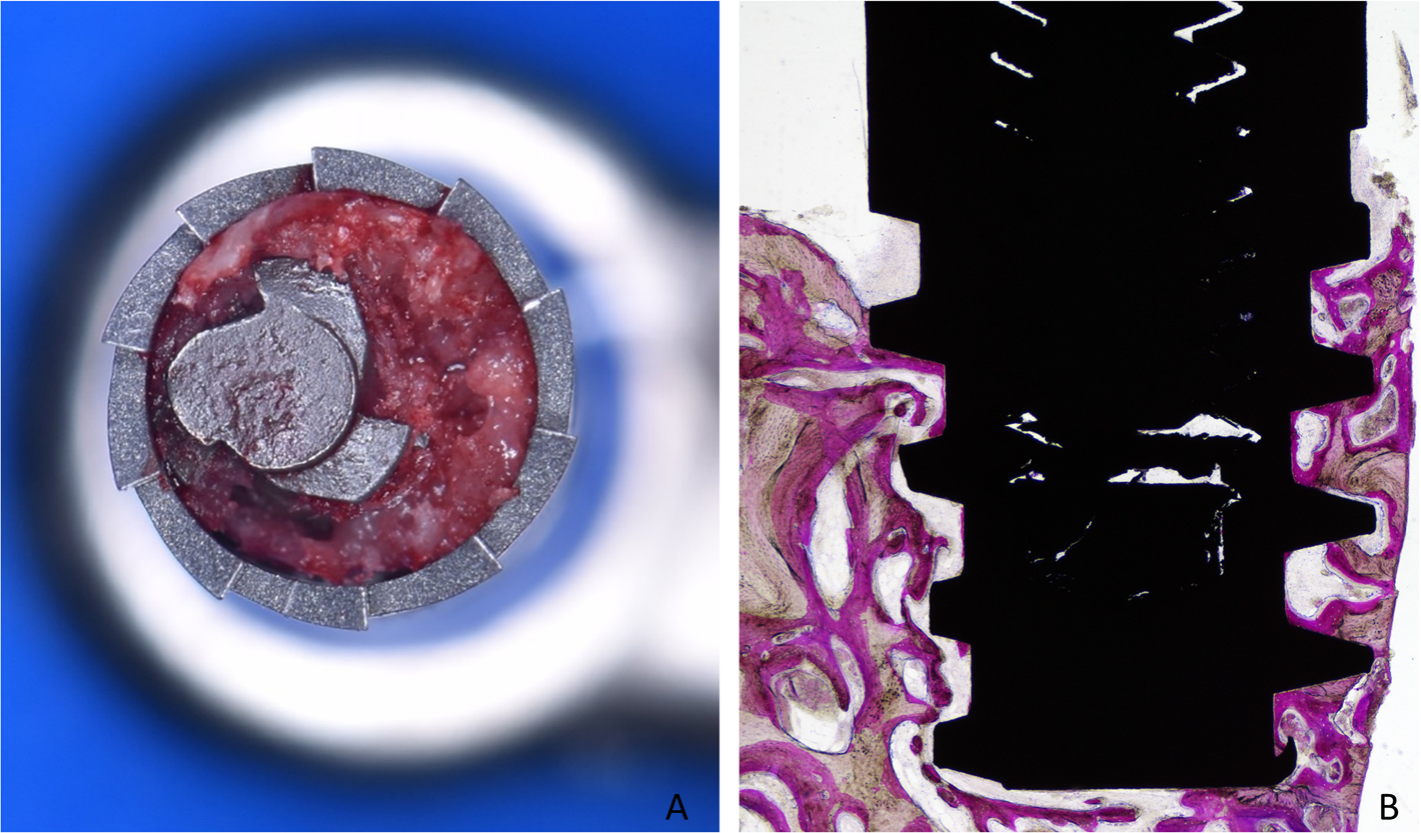
**a** Trephine used in an eccentric way to reduce the size of the biopsies. **b** Ground section illustrating the shape of the biopsies

After 8 weeks of healing, the patients were recalled to the clinic for biopsies retrieval (Fig. 2b). Full-thickness flaps were elevated, and biopsies including the mini-implants were retrieved using the trephine in an eccentric position to reduce the size of the donor site and obtain sufficient hard tissue at least at one side of the biopsies (Fig. 3) [17].

### Histological preparation of the biopsies

The biopsies were washed in saline solution and immediately stored in 10% buffered formalin. The histological process was performed in the Histology Laboratory for Hard Tissues at the University of Chieti-Pescara, Italy. All biopsies were first dehydrated in an ascending series of alcohol and subsequently embedded in resin (Technovit® 7200 VLC; Kulzer, Wehrheim, Germany). After polymerization, the biopsies were sectioned following the longitudinal axis using a precision diamond disk. Specimens of ~ 150 μm of width were obtained that were afterwards ground to ~ 30 μm of width. A staining with acid fuchsine and toluidine blue was applied.

### Histomorphometric evaluation

The histomorphometric evaluations were performed twice by an expert (KAAA), and mean values were used. The examiner was blinded, and no indications were reported on the histological slides that may have allowed the identification of groups. All histological analyses were performed in ARDEC facilities (Ariminum Odontologica, Rimini, Italy) using an Eclipse Ci microscope (Nikon Corporation, Tokyo, Japan) that was coupled with a digital video camera (Digital Sight DS-2Mv, Nikon Corporation, Tokyo, Japan). The measurements were taken using the software NIS-Elements D 4.10 (Laboratory Imaging, Nikon Corporation, Tokyo, Japan). The percentages of new bone, pre-existing (old) bone, bone debris/clot remnants, and soft tissues (marrow spaces, Haversian canals, BMUs canals) were evaluated in contact with the implant surface. All measurements were performed at × 200 magnification from the most coronal contact of bone to the implant surface (B) to the apical extension of the tissues (A). The total mineralized bone was assessed as sum of new and old bone. For the morphometrical measurements of the tissues surrounding the implant surface to a distance of 0.4 mm from the surface, the percentages of new bone, pre-existing (old) bone, bone debris/clot remnants, soft tissues (marrow spaces, Haversian canals, BMUs canals), and vessels were evaluated. A point-counting procedure was adopted, superimposing a lattice with squares of 50 μm over the histological image at a magnification of × 200.

### Data analysis

The primary variable was new bone in contact with the implant surface. New bone (bone density) around the implant surface was a secondary variable.

Mean values and standard deviations as well as 25th, 50th (median), and 75th percentiles were calculated for each outcome variable. Means, standard deviations, and 95% confidence intervals of the differences between test and control sites were calculated for each variable analyzed.

A Wilcoxon test was used to analyze differences between < 10 Ncm and ~ 30 Ncm groups. The level of significance was set at a *p* value ≤ 0.05.

## Results

**Fig. 4:**
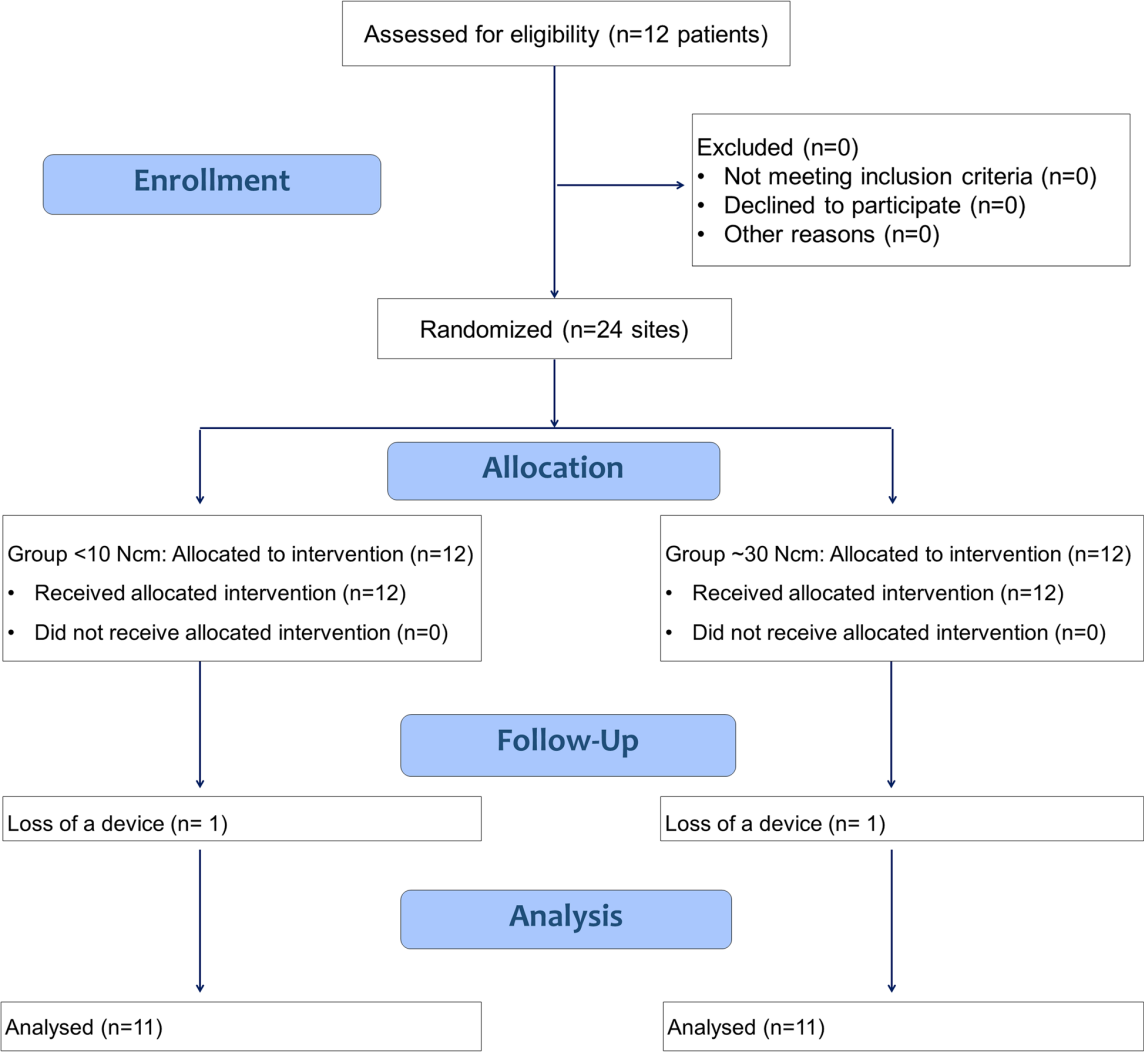
CONSORT 2010 flow diagram

Twelve subjects, four males and eight females with a mean age of 49.7 ± 10.1 years, were included in this clinical study. Eleven mini-implants were positioned in the second molar zone, 11 in the first molar zone, and two in the premolar zone of the mandible. In one participant, both mini-implants were mobile at the time of biopsies retrieval so that the implants were unscrewed without taking biopsies. No pain was reported by the patient and no suppuration or major sings of infection were detected. The patient was excluded from analysis and a total *n* = 11 was reached (Fig. 4).

**Fig. 5:**
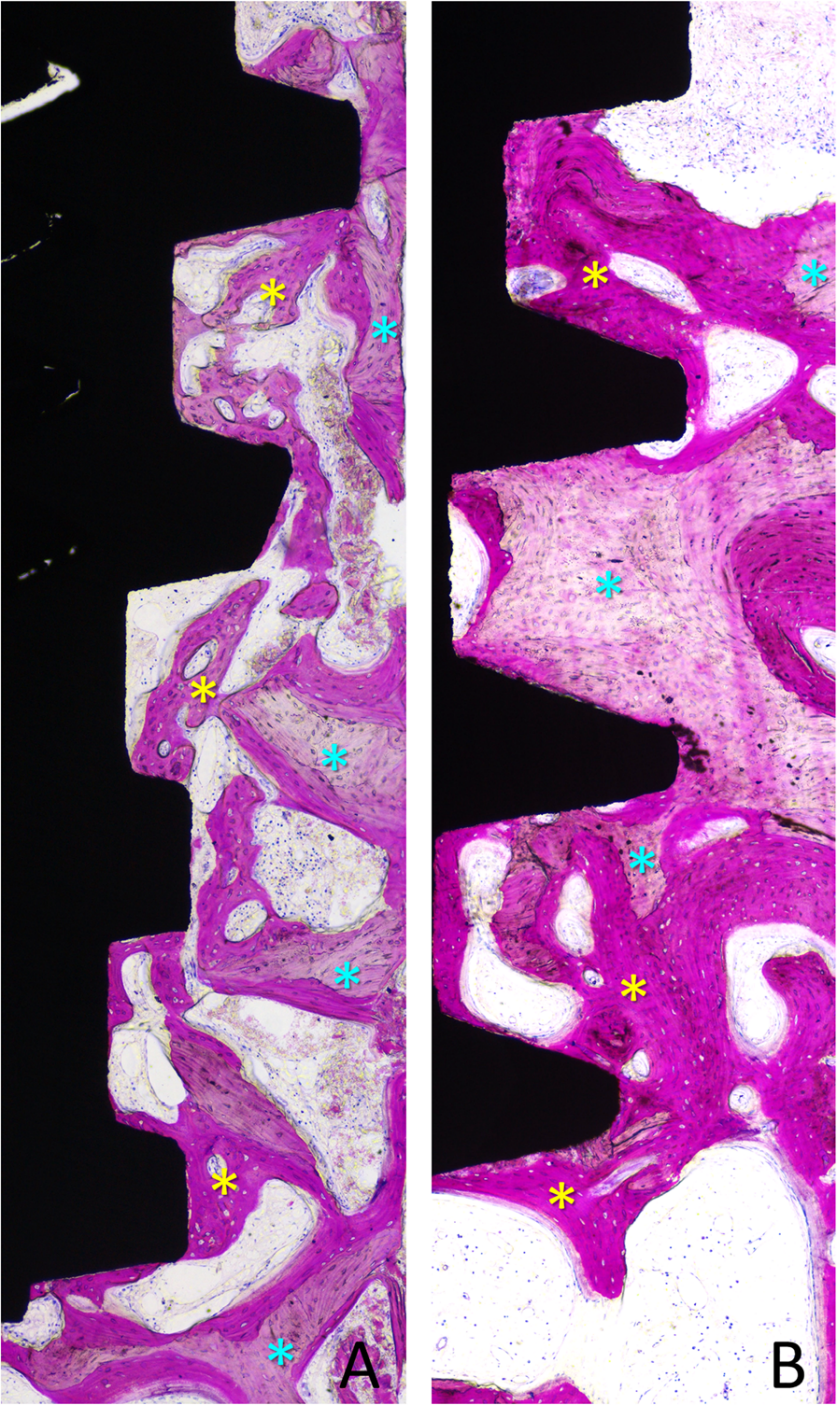
**a**, **b** Photomicrographs of ground sections of devices installed with old bone (light blue stars). Images originally grabbed at × 100 magnification. Acid fuchsine and toluidine blue stain

**Fig. 6:**
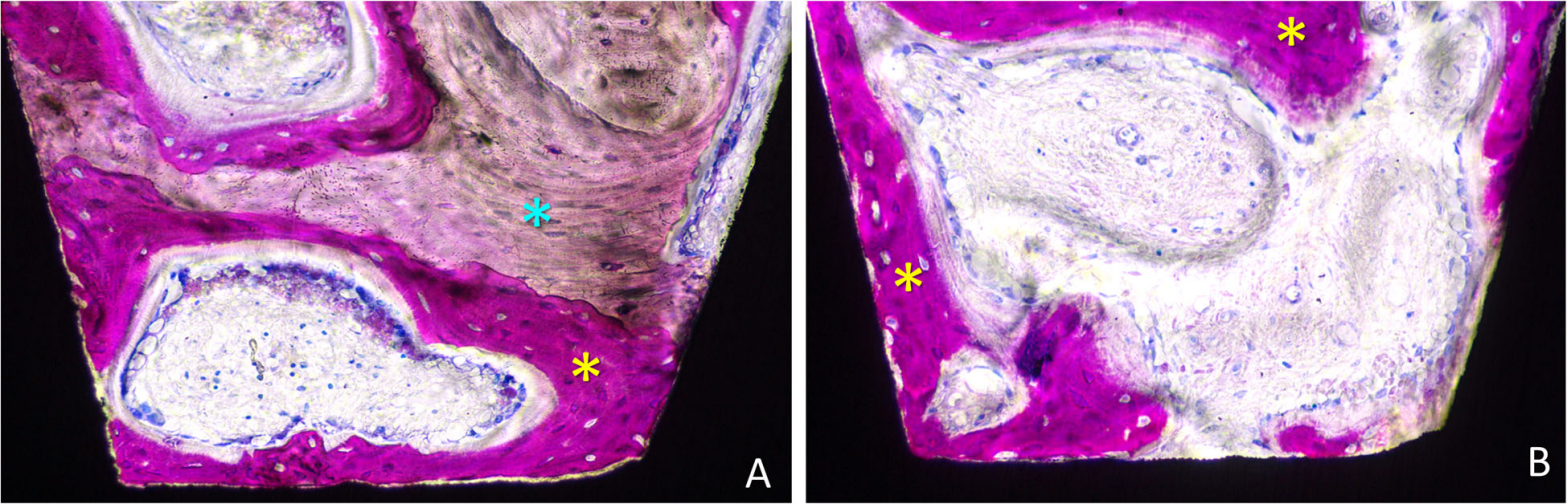
Photomicrographs of ground sections of devices installed with insertion torque (**a**) < 10 Ncm and (**b**) ~ 30 Ncm. Note newly formed bone (yellow stars) and pre-existing old bone (light blue stars) and marrow spaces. Image originally taken at × 200 magnification. Acid fuchsine and toluidine blue stain

**Fig. 7:**
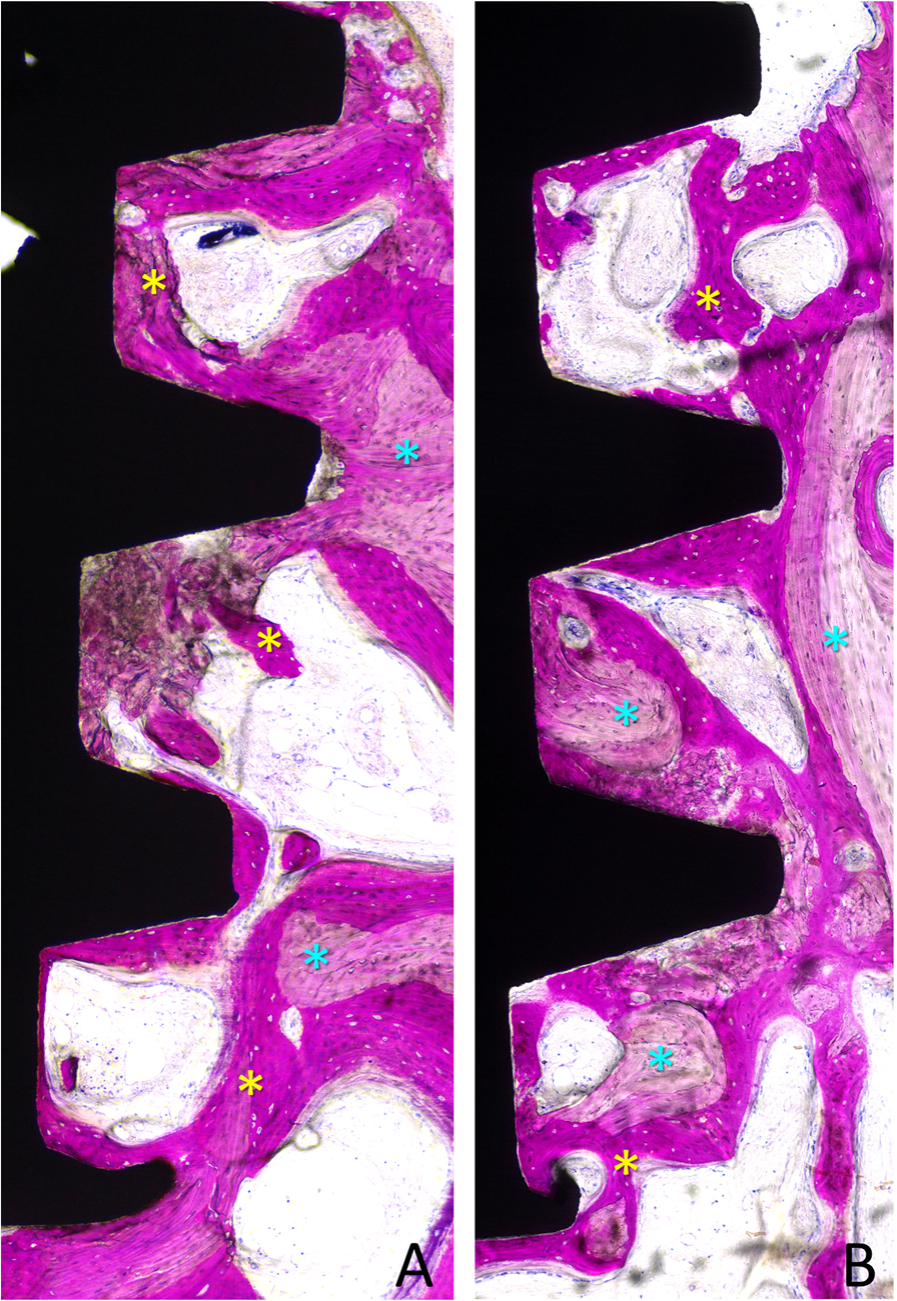
**a**, **b** Photomicrographs of ground sections of devices installed with insertion torque ~ 30 Ncm. Note newly formed bone (yellow stars) and pre-existing old bone (light blue stars). Originally grabbed at × 100 magnification. Acid fuchsine and toluidine blue stain

New bone in contact with the implant surface was found interposed between the implant and the pre-existing bone, both at the < 10 Ncm (Fig. 5 and Fig.6a) and the ~ 30 Ncm (Fig. 6b and Fig. 7) groups. The percentages reached 39.3 ± 18.5% and 49.4 ± 9.4% in the < 10 Ncm and ~ 30 Ncm groups, respectively (Table 1). The differences were not statistically significant (*p* = 0.114; Table 2). The new bone formed bridges of trabeculae connecting the pre-existing bone and the implant surface. Remnants of old bone were still found in contact with the implant surface in both groups (~ 7–8%), contributing to increase the total amount of mineralized bone to 46.8 ± 22.1% and 57.0 ± 14.1% in the < 10 Ncm and ~ 30 Ncm groups, respectively (*p* = 0.213; Table 2). Very small amounts of bone debris were detected.

**Table 1:** Percentages of tissues in contact with the implant surface after 8 weeks of healing. Mean percentages ± standard deviations (SD), medians, and 25th and 75th percentiles (25^th^; 75^th^) of tissues components on the implant surface (*n* = 11)

		New bone	Pre-existing (old) bone	Total mineralized bone	Bone debris/ clot remnants	Soft tissues
< 10 Ncm	Mean ± SD	39.3 ± 18.5	7.5 ± 9.0	46.8 ± 22.1	0.1 ± 0.2	53.1 ± 22.1
	Median (25th; 75th)	44.2 (24.2; 49.9)	4.5 (0.4; 12.9)	46.1 (35.4; 55.0)	0.0 (0.0; 0.0)	53.9 (45.0; 64.3)
~ 30 Ncm	Mean ± SD	49.4 ± 9.4	7.7 ± 7.6	57.0 ± 14.1	2.7 ± 4.7	40.3 ± 14.9
	Median (25th; 75th)	50.0 (42.7; 55.5)	5.3 (1.8; 11.5)	57.1 (48.0; 67.1)	0.0 (0.0; 3.5)	41.4 (28.1; 51.5)

None of the differences between ~ 30 and < 10 Ncm sites was statistically significant (*p* < 0.05)

**Table 2:** Percentages of tissues on and around the implant surface after 8 weeks of healing. Means, standard deviations, and 95% confidence intervals of the differences between means of ~ 30 and < 10 Ncm sites (*n* = 11)

		New bone	Pre-existing (old) bone	Total mineralized bone	Bone debris/clot remnants	Soft tissues	Vessels
Tissues in contact with the surface	Mean ± SD	10.1 ± 21.5	0.1 ± 12.2	10.2 ± 27.5	2.6 ± 4.7	− 12.8 ± 26.4	
	95% upper; lower CI	− 3.2; 23.4	− 7.4; 7.6	− 6.8; 27.3	− 0.3; 5.5	− 29.2; 3.5	
	*p* values	0.114	0.959	0.213	0.080	0.091	
Tissues around the implant surface	Mean ± SD	− 0.7 ± 14.0	− 0.1 ± 17.8	− 0.8 ± 20.3	0.9 ± 1.5	− 0.6 ± 19.6	0.5 ± 3.0
	95% upper; lower CI	− 9.4; 7.9	− 11.1; 11.0	− 13.4; 11.8	0.0; 1.8	− 12.8; 11.6	− 1.4; 2.3
	*p* values	0.859	0.929	0.790	0.066	0.929	0.508

None of the differences between ~ 30 and < 10 Ncm sites was statistically significant (*p* < 0.05)

Bone density (Table 3) was found in similar percentage in both groups for new (~ 36–37%) and pre-existing bone (~ 16%). No statistically significant differences were disclosed between the groups for any of the variables analyzed (Table 2).

**Table 3:** Percentages of tissues around the implant surface after 8 weeks of healing. Mean percentages ± standard deviations (SD), medians, and 25th and 75th percentiles (25th; 75th) of tissues around the implant surface (*n* = 11)

		New bone	Pre-existing (old) bone	Total mineralized bone	Bone debris/clot remnants	Soft tissues	Vessels
< 10 Ncm	Mean (SD)	36.6 ± 8.1	16.4 ± 11.1	53.0 ± 13.5	0.2 ± 0.5	44.6 ± 13.4	2.2 ± 1.8
	Median (25th; 75th)	38.5 (32.5; 41.5)	16.7 (8.9; 23.4)	56.6 (41.6; 60.8)	0.0 (0.0; 0.0)	40.9 (36.4; 55.5)	1.5 (1.0; 3.4)
~ 30 Ncm	Mean (SD)	35.9 ± 10.0	16.3 ± 11.4	52.2 ± 16.0	1.0 ± 1.6	44.0 ± 15.6	2.7 ± 2.2
	Median (25th; 75th)	37.2 (29.6; 42.8)	11.7 (9.4; 23.9)	55.2 (39.7; 62.8)	0.0 (0.0; 2.0)	42.5 (32.3; 57.8)	2.3 (1.3; 3.4)

None of the differences between ~ 30 and < 10 Ncm sites was statistically significant (*p* < 0.05)

## Discussion

The aim of the present study was to evaluate histomorphometrically the healing at implants installed with standard or very low insertion torque values. Values of < 10 Ncm at the test and ~ 30 Ncm at the control sites were obtained. A higher amount of osseointegration was found at the ~ 30 Ncm compared to the < 10 Ncm torque, even though a statistically significant difference was not achieved.

In the present study, implants installed with low insertion torque integrated into the newly formed bone. This outcome is in agreement with other studies that reported similar results.

In an experiment in dogs [5], implant sites were prepared in such a way to obtain insertion torque of ~ 70 Ncm, ~ 30 Ncm, or close to 0 Ncm. The highest amount of bone-to-implant contact was observed at the ~ 30 Ncm implants at which ~ 7% and ~ 2–3% higher osseointegration was found compared to the close to 0 sites and ~ 70 Ncm groups, respectively. The highest bone density, however, was found around implants of the ~ 70 Ncm group. It was concluded that implants installed in sites with very low insertion torque may osseointegrate similarly to the implants installed with higher torque.

In another experiment in dogs [4], the bone healing at implants installed with insertion torque of about 7, 15, 20, and 35 Ncm was evaluated. The highest values of osseointegration and bone density were found at implants with torque ~ 35 Ncm, being the bone-to-implant contact ~ 12% higher compared to that observed at the sites with torque < 10 Ncm. The outcomes from these two studies [4, 5] are in agreement with the results from the present study that reported ~ 10% of more mineralized bone at the ~ 30 Ncm compared to the < 10 Ncm torque.

In another experiment [1], the healing at implants installed in the mandible of sheep using the torque of ~ 10 Ncm or ~ 110 Ncm was evaluated after 1, 2, 3, 4, and 6 weeks. Higher osseointegration and higher removal torque were seen at the high torque group in all periods of observation. Moreover, after 6 weeks of healing, 40% of the pre-existing bone was found remodeled in the high torque group, while only 15% of newly formed bone was found in the lower torque group. It was concluded that a very high insertion torque in dense cortical bone did not engender necrosis or implant failure, and increased bone stability.

Nevertheless, in a consensus conference [3], it was recommended to use implant insertion torque included between 20–45 Ncm for immediate single tooth loading. This statement is also supported by an experimental study in dogs in which implants inserted with a torque of either 30 Ncm or 70 Ncm were immediately loaded at the test sites or left unloaded at the control sites [6]. Higher bone-to-implant contact and bone density were found at the loaded compared to the unloaded implants. Moreover, a higher bone-to-implant contact was found at the 30 Ncm group compared to the 70 Ncm group. This last outcome is corroborated by the studies above mentioned [4, 5]. This, in turn, means that it may be not necessary to obtain torque > 30 Ncm also in the case of immediate loading, assuming that splinted reconstructions are provided [2].

Even though the present study showed that implants inserted with a low torque may osseointegrate properly, it does not give any support regarding the long-term stability. It has to be considered that, in clinical studies, a lower survival rate was found at implants which presented no rotational primary stability compared to implants presenting primary rotational stability [14, 15]. This has to be kept in mind when a primary rotational stability cannot be achieved at implant installation. Nevertheless, when a stability is obtained, optimal results might be obtained also in a long-term prospective.

In a clinical study [18], 40 implants, 6 mm long, were installed in the posterior region of the jaws and loaded with single crowns after 6–7 weeks of non-submerged healing. The implants had a diameter of 4.1 or 4.8 mm. Eighteen implants presented an insertion torque of ≤ 15 Ncm. Two implants with a diameter of 4.1 mm were lost before loading in two heavy smokers patients. One implant presented a torque ≤ 15 Ncm and was installed in bone of quality 4, while the other implant presented a torque > 35 Ncm and was installed in bone of quality 1. Non further losses were registered during the 5 years of follow-up.

In a clinical study [19], 30 6 mm and 30 10 mm long implants were restored with single crowns after 6–7 weeks from installation. All implants had a diameter of 4.1 mm. Eighteen of the 6 mm and 11 of the 10 mm long implants presented an insertion torque < 15 Ncm. After 5 years of loading, three of these low insertion torque implants were lost in the 6-mm group, one before loading and two after some years of function. No < 15 Ncm implants were lost in the 10-mm group. From these two long-term studies, it can be assumed that the length and diameter of the implants as well as the bone quality might influence healing and function. Also in the present study, more implants were lost at the low compared to the standard insertion torque sites. However, the small dimensions of the implants, concomitantly to the non-submerged healing applied, may have influenced this outcome.

A rotational loss of stability can be also found after healing, i.e., before loading. In a clinical study, after 6 weeks of healing, four implants out of 132 presented a slight rotation at abutment connections and were left unloaded for further 6 weeks [20]. After this period, no rotational instability was found, and the prosthetic restoration could be completed. In another clinical study, abutment connection was performed after either 1 week or after 5 weeks from implant installation [21]. Two implants of the 1-week group and one of the 5-week group were found with rotational instability. These implants were left to heal for further 12 weeks and, finally, a good rotational stability was achieved so that the prosthetic rehabilitation could be performed.

In the present study, after 8 weeks of healing, new bone was found at a percentage ranging from 39.3% to 49.4%. It has been shown that when a longer period of healing was allowed, osseointegration increased in percentages at implants with a surface similar to that used in the present study. Percentages ranging from about 63% to about 85% after 3–4 months of healing were reported in human studies [22–24]. Several animal studies were also performed using implants with the same surface used in the present study. However, it has to be noted that in those animal experiments, similar amounts of osseointegration (46.1% to 50.6%) to those observed in the present study were found after 1 months of healing [25, 26]. This faster osseointegration in animals compared to humans has been described in a report that considered several factors that may influence osseointegration [27].

In the present study, pre-existing bone was still present in contact with the implant surface after 8 weeks of healing. Several humans [22, 23, 28] and animal studies [25, 26] have reported the presence of old bone in the early stages of healing, even after 3–4 months from implant installation, however, in very low percentages.

In the present study, the macrogeometry of the implants was designed to increase the resistance to the deformation during the insertion. It was selected the geometry of a transmucosal implants, choice supported by the similar outcomes shown in marginal bone levels between submerged and non-submerged healing [29].

The main limitation of the present study was represented by the small sample. Moreover, one subject was excluded from the analyses due to failed implants so that the sample was reduced from 12 to 11.

From the data of the present study, it can be concluded that a trend of higher osseointegration was observed at the ~ 30 Ncm compared to the < 10 Ncm torque group. Nevertheless, it can be concluded that the implant installed with a very low torque may achieve a good integration.

From a clinical point of view, considering the lower osseointegration seen at the low-torque implants, in the case of poor bone quality, an under-preparation of the recipient sites should be applied, and longer and wider implants should be installed. Moreover, in the presence of a low insertion torque, applying a sub-merged healing protocol might be advisable, as well as a delayed loading.

## Data Availability

The datasets used or analyzed during the current study are available from the corresponding author on reasonable request.
